# Exploring Young Adults' Attitudes Toward AI-Driven mHealth Apps: Qualitative Study

**DOI:** 10.2196/76075

**Published:** 2025-09-26

**Authors:** Ali Aboueldahab, Gabriele Damaschi, Marco D'Addario, Patrizia Steca

**Affiliations:** 1 Department of Psychology, University of Milano-Bicocca, Piazza dell’Ateneo Nuovo, 1, Milano, 20126, Italy, 39 02 6448 1

**Keywords:** AI-driven mHealth apps, user attitudes, thematic analysis, perceived risks, usability, innovation and reliability, emotional interaction, privacy concerns, mobile health, artificial intelligence

## Abstract

**Background:**

Artificial intelligence (AI)–driven mobile health (mHealth) apps are emerging as a promising tool for health management, yet little is known about users’ psychological perceptions and attitudes toward these technologies. Understanding these aspects is crucial for both the appropriate design and the effective use of these technologies, ensuring the psychological and physical well-being of potential end users.

**Objective:**

This study aimed to investigate the attitudes and perceptions of young adults toward a possible use of AI-driven mHealth apps, focusing on the perceived benefits and potential concerns related to their future adoption.

**Methods:**

A qualitative focus group methodology was used. Fifteen participants (12 men, 3 women; mean age 27 years, range: 25‐34 years) were recruited. Data were analyzed using thematic analysis to identify key themes influencing engagement with these technologies.

**Results:**

Four main themes emerged: “Usability,” which emphasized the importance of user-friendly, personalized experiences; “Innovation and Reliability,” where participants expressed both enthusiasm and skepticism towards AI’s potential; “Affectivity and Interaction with AI,” highlighting mixed opinions on the emotional impact of AI interactions; and “Perceived Risks,” which focused on concerns regarding data privacy and the need for human supervision. These factors contributed to ambivalent attitudes toward AI-driven mHealth apps, with some participants being open to adoption, while others remained cautious.

**Conclusions:**

To foster greater engagement with AI-driven mHealth apps, developers should prioritize usability, trust, emotional support, and privacy issues, considering users’ psychological needs and expectations. The findings offer valuable insights for designing more user-oriented mHealth solutions. Further research should explore how perceptions evolve with direct experience and long-term use.

## Introduction

In recent years, the rapid advancement of artificial intelligence (AI) has had a profound impact on various sectors of society, shaping industries, decision-making processes, and everyday experiences [1-3]. One of the most significant developments has been the rise of generative AI models, such as ChatGPT, Midjourney, and Gemini, which have gained widespread attention for their ability to generate human-like text, images, and other creative outputs. This rapid proliferation of AI-driven tools has sparked discussions about their integration into multiple aspects of daily life, from education and entertainment to professional services and health care [4].

Among these domains, health care has emerged as a particularly critical area for AI innovation, with growing interest in leveraging AI technologies to improve medical diagnostics, treatment recommendations, and patient support systems [5-7]. A specific and increasingly relevant subset of AI applications within health care is mobile health (mHealth) [8]**—**digital platforms designed to support health-related services and patient engagement via smartphones and other mobile devices. mHealth apps have gained popularity due to their accessibility and convenience, enabling users to track their health metrics, receive medical advice, and engage with health care providers remotely [9].

The integration of AI into mHealth apps is gaining momentum as developers seek to enhance functionalities through AI-powered tools, such as chatbots for patient interaction, symptom checkers, and personalized health recommendations [10,11]. In this context, AI-driven mHealth apps—defined as mHealth platforms that integrate AI-based tools to process health data, provide interactive support, and deliver tailored recommendations [8]— are emerging as a new frontier in digital health care. However, given the current state of development, only a small number of users have had a direct experience with these beta versions that incorporate AI-driven features. This limited exposure reflects the early stages of these apps and the challenges in widespread implementation. Despite this momentum, the introduction of AI in such a sensitive domain as personal health management raises several concerns and uncertainties [12,13]. Unlike traditional health care solutions, AI-driven mHealth apps rely on machine learning algorithms, raising concerns about privacy, security, transparency, and potential biases. While these technologies offer promising advancements, current discussions in the literature and public discourse reveal a landscape marked by both enthusiasm for innovation and apprehension regarding ethical, technical, and social implications [14,15].

One of the key gaps in the literature concerns the public perception toward the integration of AI within mHealth apps. While previous studies have explored the technical feasibility and the general acceptance of AI in health care settings, including hospitals and clinical diagnostics, there is limited research focusing specifically on mHealth apps incorporating AI-powered tools—such as interactive chatbots handling personal data and health-related information—and the way everyday users perceive their benefits and risks [16-18]. Given that these apps are designed to operate in highly personal and private contexts—potentially serving as a first point of contact for health-related inquiries—it is crucial to explore what expectations, concerns, and attitudes individuals hold toward them, even in the absence of direct experience or exposure to these tools, and what factors influence their acceptance or skepticism [19]. Unlike AI adoption in traditional medical environments, where health care professionals act as intermediaries, mHealth AI tools are envisioned to function autonomously, making user trust and acceptance even more critical key factors to investigate [20].

Existing research on AI acceptance has examined factors like trust, perceived usefulness, and data privacy concerns [12,14,21], but studies specifically addressing these aspects within mHealth apps remain limited. Given the relatively early stage of AI implementation in mHealth, understanding public attitudes is crucial for shaping ethical, effective, and user-centered AI applications in health care. Issues related to data privacy, trust in AI-generated recommendations [12], potential biases in AI algorithms [19], and the perceived reliability of AI-driven health tools remain underexplored in relation to users’ willingness to adopt such innovations [6].

Given the novelty of AI integration in mHealth, this study specifically examines young adults (25‐35 years old) as a strategically relevant demographic. This cohort represents both the primary users of digital health technologies [22-24] and digital natives who are more likely to engage with emerging AI interfaces compared with older aged groups [25,26]. Focusing on this population helps minimize age-related technology adoption biases that might confound perceptions in older groups, while providing insights from individuals most likely to shape early adoption patterns.

Against this backdrop, this study aims to explore the key factors shaping individuals’ attitudes toward the integration of AI tools within mHealth. Specifically, it investigates perceived risks, doubts, and concerns, as well as potential benefits and innovations that users associate with AI-powered health technologies.

The remainder of this article is structured as follows: the next section outlines the materials and methods used in this study, detailing the research design, data collection process, and analytical framework. This is followed by a comprehensive presentation of the results, highlighting key findings related to public perception, concerns, and expectations. The discussion section then interprets these findings in the context of existing literature, drawing comparisons with previous studies and identifying potential implications for developers, health care professionals, and policymakers. Finally, the conclusion summarizes the primary insights derived from this research and outlines possible directions for future studies in this rapidly evolving field.

## Methods

### Study Design

The study used a qualitative research approach based on focus group discussions. Two focus groups were conducted in January 2025 to explore participants’ perspectives, experiences, and opinions on the topic. Focus groups were chosen as they foster discussion, allowing for the emergence of insights that might not surface in individual interviews [27]. This format enables participants to engage with one another, facilitating a more comprehensive understanding of shared and divergent viewpoints [28,29].

### Ethical Considerations

The study design, procedures, and informed consent form were evaluated and approved by the local commission for minimal-risk studies of the Psychology Department of the University of Milan-Bicocca (protocol number RM-2024‐854). Participants did not receive any compensation for their involvement.

### Study Participants

The study involved 15 participants (12 males and 3 females) aged 25 to 34 years old. The first focus group included 7 participants (6 males and 1 female) aged 25 to 28 years old, while the second group consisted of 8 participants (6 males and 2 females) aged 25 to 34 years old. Participants were selected through a snowball sampling method via messaging apps (eg, WhatsApp [Meta]) to ensure diversity while maintaining relevance to the research topic. Each session lasted approximately 120 minutes.

Eligibility criteria required participants to: (1) be young adults or digital natives (18-35 years old), (2) have varying levels of familiarity with mobile health (mHealth) apps and AI, assessed through a preliminary question during recruitment, and (3) be over the legal age. The participants were evenly distributed between the 2 groups based on their levels of familiarity with and usage of mHealth apps and AI, in order to create as homogeneous groups as possible and encourage a more productive discussion.

While recruitment efforts sought a balanced sample, the final composition resulted in a gender imbalance (12 men vs 3 women), primarily due to participant availability and recruitment challenges. This limitation is further addressed in the discussion section, where its potential impact on the findings is considered, along with other constraints of this study.

### Procedure

Each focus group followed a structured discussion guide to ensure consistency while allowing for open-ended exploration of key themes. The sessions were moderated by a researcher and lasted approximately 120 minutes. The focus groups were conducted remotely via WebEx, ensuring accessibility for all participants.

At the beginning of each session, participants were welcomed and thanked for their participation. The moderator briefly introduced himself and provided an overview of the study’s objectives. A general definition of mHealth apps was presented, outlining their main types and uses. The concept of a focus group was explained, including its purpose and ground rules, such as mutual respect, open expression of opinions, and the absence of right or wrong answers. Participants were reassured that their insights were valuable and encouraged to share their perspectives freely.

To create a comfortable and engaging atmosphere, the discussion began with an icebreaker activity, where participants introduced themselves by sharing their names, age, and professional background. This step aimed to foster interaction and establish a collaborative dynamic. The discussion proceeded with a series of structured interactive exercises. First, a free association exercise (mHealth apps), in which participants were asked to list 3 words that came to mind when thinking about mHealth apps. Their responses were visualized in a word cloud using Mentimeter. Each participant then explained their choices, and an open discussion followed to explore shared and contrasting views. Second, anchored association exercise (mHealth apps and AI); the previous exercise was repeated, this time focusing on AI-driven mHealth apps. Participants provided 3 words, which were compiled into another word cloud. The discussion centered on emerging themes and differences in perception between traditional and AI-enhanced mHealth apps. Third, a role-playing scenario (using an AI-integrated mHealth app), where participants were asked to imagine using an AI-integrated mHealth app and to identify one advantage and one disadvantage of such technology. Their responses were collected, and a group discussion was conducted to explore the potential benefits and challenges of AI in health care apps. Fourth, preferred communication style for AI chatbots; participants described their ideal AI chatbot communication style by selecting three descriptive words, which were visualized in a word cloud. They then explained their choices, leading to a broader discussion on expectations and preferences regarding AI-driven health communication. Fifth, ranking exercise (AI chatbot communication features), in which participants were asked to rank the words from the previous exercise in order of importance, reflecting their priorities for chatbot interaction. The final group ranking was analyzed collectively, and participants shared their reflections on the results. The focus groups concluded with a summary of key discussion points, followed by a debriefing on the study’s objectives and the intended use of the collected data. Participants were invited to ask any final questions or provide additional comments before the session ended.

### Data Analysis

The 2 focus group sessions were audio-recorded and subsequently transcribed. The transcripts were analyzed qualitatively using inductive thematic analysis, following the 6-phase approach outlined by Braun and Clarke [29]: (1) familiarizing with the data, (2) generating of initial codes, (3) searching for themes, (4) reviewing themes, (5) defining and naming themes and, (6) producing the report. This analytical approach is inherently recursive rather than strictly linear, allowing for flexibility in revisiting and refining earlier phases as new insights emerge. Thematic analysis was chosen as it enables an in-depth exploration of participants’ perspectives, capturing both shared viewpoints and individual divergences, thereby facilitating a nuanced understanding of the topic under investigation.

To ensure a rigorous and unbiased analytical process, 2 researchers independently reviewed the transcripts multiple times, noting initial impressions and generating preliminary codes. This parallel approach was intended to minimize researcher bias and provide a broader interpretative perspective. The degree of agreement between the 2 researchers was calculated directly, resulting in an agreement rate of approximately 82.76%, indicating a very high level of consistency. After calculating the agreement, the researchers presented their identified codes, explaining the rationale behind their choices and highlighting areas of consensus and divergence. Through discussion, they worked toward a shared interpretation, reconciling differences where necessary. This collaborative process enabled the researchers to refine the thematic structure, identifying the main themes for the thematic analysis.

Once the themes were clearly defined and named, representative verbatim excerpts were selected and organized in a table, alongside their corresponding codes and main themes. Data were analyzed in their original language to preserve participants’ intended meanings, while coding and theme development were conducted in English. The selected transcript examples were translated into English for reporting purposes.

## Results

### Overview of the Findings

The participants were divided into the 2 groups considering their levels of familiarity with and usage of mHealth apps and AI. Specifically, 8 out of 15 (mean 53.3%, SD 12.9%) reported frequent use or a positive attitude toward mHealth apps, while 7 out of 15 (mean 46.7%, SD 12.9%) used them less often or not at all. Similarly, all participants had used AI at least once, with 9 out of 15 (mean 60.0%, SD 12.6%) being frequent users, while the remaining 6 (mean 40.0%, SD 12.6%) used AI sporadically or were opposed to it.

Four themes were identified in relation to the research question: “Usability,” “Innovation and Reliability,” “Affectivity and Interaction with AI,” and “Perceived risks.” The “Usability” theme included 3 subthemes: personalization, accessibility and cost, and AI communication style. The “Innovation and Reliability” theme encompassed subthemes related to reliability and understanding, perceived innovation and trust in technology, and use as a monitoring tool. The “Affectivity and Interaction with AI” theme explored the emotional and interactive dynamics with AI, with subthemes of psychological impact and human-AI interaction. Finally, the “Perceived Risks” theme addressed concerns about data privacy and management, the need for human supervision, and uncertainties surrounding the use of AI in mHealth apps, with subthemes including privacy and data security, human oversight and control, and uncertainty and knowledge gaps (refer to Table 1 for a brief description of the themes and subthemes).

**Table 1.** Summary of the themes and subthemes that emerged from the thematic analysis.

Themes and subthemes	Description
Usability	This theme addresses how participants perceive the features of mHealth^a^ apps with AI^b^, focusing on aspects like personalization, accessibility, and AI communication style.
Personalization	The extent to which AI adapts to individual needs, progress, and personal data, offering tailored support and interactions.
Accessibility and cost	Reflections on how easy it is to access and use these apps, along with considerations of their affordability.
AI communication style	Preferences regarding the tone, manner, and ideal interaction style of the AI when addressing users.
Innovation and Reliability	This theme explores the level of trust participants have in mHealth apps with AI, their perception of innovation, and how these technologies are used.
Reliability and understanding	How reliable participants perceive AI to be in providing accurate and useful responses.
Perceived innovation and trust in technology	How participants perceive AI as either a necessary advancement or a technology to be approached with skepticism.
Use as monitoring tool	Reflections on the role of mHealth apps with AI primarily as tools for tracking personal health data.
Affectivity and Interaction with AI	This theme examines the emotional dynamics, uncertainties, and interactions between users and AI in mHealth apps.
Psychological impact	Emotions evoked by interacting with an AI chatbot in a health context, including motivation, anxiety, trust, and skepticism.
Human-AI interaction	Reflections on the comparison between AI chatbots and human operators, highlighting the advantages of personalization and constant availability, but also the need for human interaction for trust and understanding.
Perceived Risks	This theme addresses concerns related to data privacy, the need for human oversight, and uncertainties regarding the use of AI in mHealth apps.
Privacy and data security	Concerns about who manages the collected data, the level of access AI has to personal information, and the differences between traditional health apps and those with AI.
Human supervision and control	The need for human oversight to ensure safety, particularly in managing sensitive health data and situations.
Uncertainty and knowledge gaps	Difficulties in understanding how AI-powered apps work, concerns about the technology, and broader issues such as environmental impact.

a mHealth: mobile health.

b AI: artificial intelligence

### Usability

#### Overview

Participants emphasized the practical advantages of AI-driven mHealth apps, particularly their precision, ease of use, and ability to deliver personalized, real-time support that enhances user experience. In addition, affordability was identified as an important factor that might influence the adoption of such technologies. However, opinions diverged regarding the preferred communication style, with some imagining a preference for a formal and direct approach, while others anticipated a more friendly and engaging interaction.

*Like, just thinking about AI doing an initial screening for acceptance—it could speed things up. Or you ask it something, and it gives you an answer in 30 seconds instead of having to book an appointment six months later. But that’s only if it’s really accurate. I don’t know, maybe for very basic things. I’m not sure where the line is between what you can actually ask a chatbot in an app and what really needs a doctor in a proper visit.*

[Female #1]

*So, it turns out to be more precise and easier to use compared to a regular app—more targeted and more user-friendly. I think this would make people more likely to use it.*

[Male #8]

*Artificial intelligence offers more engaging suggestions compared to a regular application that lacks this feature.*

[Male #10]

#### Subtheme 1: Personalization

A major perceived advantage of AI-driven mHealth apps was thought to be their ability to provide a highly personalized experience. Participants highlighted the value of dynamic personalization, imagining apps that refine recommendations based on progress and align with individual needs and goals to enhance engagement. This dynamic personalization was considered a key differentiator from traditional mHealth apps.

*Personalization is the key because, imagining this app, I see a section where I can specify my interests, what I enjoy doing, and my goals. […] In my view, the main advantage of artificial intelligence in this context is a better match between user interests and objectives […] For example, I imagine an app that suggests hiking trails—places I wasn’t aware of but that, thanks to artificial intelligence, it can recommend based on my activity level and the preferences I’ve set.*

[Male #7]

*The ability to customize a tool definitely adds value—it can do the work for us, access more data, process it, and provide what I’d consider a better answer.*

[Female #2]

#### Subtheme 2: Accessibility and Cost

Participants valued the immediacy of AI-powered responses, considering them a key advantage for health and fitness management. However, some raised concerns that the speed of response might compromise the depth and accuracy of the provided insights. Given these potential limitations, participants agreed that these services should ideally be financially accessible, ensuring that they could remain a cost-effective alternative to professional consultations.

*The cost is a concern—it shouldn’t be more expensive than consulting a nutritionist, that’s for sure.*

[Male #9]

*The ability to receive immediate information is always a strong point.*

[Male #2]

*Speed is crucial—the fact that I don’t have to wait for a human response would be a fundamental advantage.*

[Male #7]

#### Subtheme 3: AI Communication Style

There was no consensus on the preferred communication style for AI-powered chatbots, which are considered a key tool for the envisioned AI-driven mHealth apps in this study. Some participants favored a pragmatic, direct approach—emphasizing clarity and efficiency in responses—could be more effective. Others preferred a friendlier, more conversational tone, which might be preferable, especially when discussing sensitive health-related topics, where the ideal tone might depend on context. While a professional, detached tone could enhance credibility in medical discussions, a warmer and more engaging approach could be better suited for fitness or lifestyle guidance.

*It should be pragmatic—it needs to provide concise and direct answers to the issues I bring up.*

[Male #10]

*(it should be) Formal and straightforward, because I’m not looking for casual chat or small talk. If I use an app or chatbot, I don’t need friendliness—I just want something fast, efficient, and simple, without being overly technical.*

[Female #1]

*I don’t think I’d want a friendly interaction. In fact, that kind of interaction bothers me.*

[Male #12]

*I’d like the chatbot to address me in a friendly manner, but not as if it were a doctor or an ultimate expert trying to teach me how to live.*

[Male #4]

*Whatever role this bot is designed to take on, I believe each role requires a different communication style. If it is meant to provide medical advice, it should, like human doctors, maintain a professional and detached approach to preserve its credibility.*

[Male #1]

### Innovation and Reliability

#### Overview

Participants expressed mixed opinions regarding the reliability of AI-driven mHealth apps, particularly in terms of the accuracy of the information these apps might provide. While some recognized their potential, others emphasized the need to verify AI-generated insights before relying on them. Trust in AI technologies varied among participants, with some seeing it as an intriguing and potentially valuable tool for health management, while others remained skeptical about their consistency and applicability across different health-related contexts.

*I don’t mind if the app has a deeper understanding of me—this doesn’t scare me; on the contrary, it intrigues me. In a way, it even adds a bit of creativity to using the app.*

[Male #11]

*How reliable is the information provided by artificial intelligence? It might be useful in certain areas, but I’m not sure it’s equally reliable across all applications.*

[Male #3]

#### Subtheme 1: Reliability and Understanding

A recurring concern among participants was the degree of trust they might place in AI-generated responses. While some recognized advancements in AI technology, they remained cautious about the possibility of errors or misleading recommendations. Several participants highlighted that the reliability of these apps could depend on the sophistication of the AI model used, acknowledging the significant progress made in 2025. Despite these improvements, many participants indicated that they would still prefer consulting a human expert, particularly when dealing with complex or highly personalized health concerns—a topic that will be further explored in relation to theme 4: perceived risks, subtheme 2: human supervision and control.

*A chatbot can make serious mistakes. Trusting a bot instead of a human is risky—of course, humans can also make errors, but a person can look you in the eye and read between the lines, whereas a bot operates in a more uncontrolled manner.*

[Female #2]

*The risk is that it might promote certain behaviors that are not suitable for everyone.*

[Male #12]

*The chatbot’s level is important. If we’re talking about something as advanced as ChatGPT or Gemini, I think we’ve reached a very high standard by 2025.*

[Male #9]

#### Subtheme 2: Perceived Innovation and Trust in Technology

Participants expressed divergent views on the role of AI in health care, oscillating between enthusiasm for its potential and skepticism about its long-term impact. Some perceived AI as a temporary trend, doubting that its applications would revolutionize health management in a meaningful way. Others, however, regarded AI as an evolving tool with growing significance, predicting that it would become increasingly integrated into daily life and professional settings.

*I wrote down* “*innovation” and* “*trend” … But to be honest, I have some doubts about calling it “innovation”—I see it as more of a trend, in my opinion.*

[Male #5]

*I don’t agree with calling it a trend. Even though AI is experiencing a huge boom right now, it’s being used everywhere, and I don’t think it’s just a passing fad. I believe AI is here to stay, to improve further, and, in a way, to increasingly permeate our lives.*

[Male #4]

#### Subtheme 3: Use as Monitoring Tool

One of the most frequently mentioned applications of AI-driven mHealth apps was their role in health monitoring, with participants seeing AI tools as a way to enhance traditional mHealth apps they were already familiar with. These apps were envisioned as valuable for tracking health parameters like medical health, sleep patterns, nutrition, and physical activity, offering the ability to record, manage, analyze, and visualize personal data, especially when combined with AI-driven personalization and real-time feedback.

*What I like most about these apps [traditional mHeatlh apps] is their ability to track progress. It’s not so much about receiving notifications to remind me to move […] I would appreciate If they provided me an overview of how things have been going over time—whether there have been improvements or declines.*

[Male #11]

*I used an mHealth app for running, mainly for tracking my progress. […] If the app included also an AI chatbot that guides my training and suggests specific workouts it would be even easier.*

[Male #5]

*The fact that an app can monitor my physical condition, diet, and habits is really convenient. I can see changes over time and adjust my habits immediately.*

[Male #4]

### Affectivity and Interaction With AI

#### Overview

The focus groups highlighted various concerns and reflections regarding the psychological implications of interacting with AI-driven mHealth apps, especially if chatbot-based consultations were to replace human specialists. Participants expressed mixed feelings about this potential shift, recognizing both potential benefits and drawbacks.

*Anxiety and motivation go hand in hand. I haven’t used these apps [mHealth traditional apps] much, but when I did, they always started with motivation but ended up making me anxious.*

[Male #2]

*If it’s about something sport-related—like asking the chatbot how many kilometers I should run to train for something—that’s fine. But if it’s a deeper health-related conversation about symptoms or medical issues, it would feel strange to discuss it with a chatbot. I would find it a bit distant.*

[Female #1]

#### Subtheme 1: Psychological Impact

From an emotional perspective, participants described a range of potential psychological effects associated with these apps. Some raised concerns about increased stress levels, particularly due to frequent health-related notifications, which could inadvertently heighten anxiety rather than alleviate it. Participants also raised concerns about how AI-generated recommendations might affect motivation and emotional well-being, suggesting that the absence of human interaction could reduce accountability or increase stress in some users. Conversely, several participants viewed AI-driven support as an opportunity for greater personal empowerment. In this regard, they suggested that receiving recommendations from an app—without the perceived pressure of a direct human interaction—might foster a sense of autonomy and reduce stress.

*I already don’t sleep much. If I had an app telling me* “*It’s 8 PM, time to go to bed,” I’d get even more anxious, and, in the end, I’d probably stay up until 3 AM.*

[Female #3]

*My fear is that if this AI provides recommendations, support, and guidance, I might lose autonomy—especially regarding diet and exercise. Since the chatbot is always available, it’s different from dealing with a professional.*

[Male #11]

*Thinking of something that is a pro and con at the same time... For example, besides sports, another thing mentioned earlier is the area of diets, meal plans, etc. Maybe you don’t feel the anxiety of saying,* “*Oh no, I didn’t stick to this,” if you just report it to an app. Indicating that you didn’t meet a certain goal doesn’t come with the fear of judgment or reactions. [...] But the downside is that you might lack some motivation, because you think,* “*Who cares if I write on my phone that I binged on sweets?”—compared to having to admit it to a real person, like a professional you’re seeing.*

[Female #1]

#### Subtheme 2: Human AI Interaction

The nature of the interaction between humans and AI was another key theme that emerged. Some participants reported that chatbot-based consultations could potentially lack warmth, emotional support, and the relational aspects that characterize human interactions, particularly in health care settings. This perceived absence of a human element was viewed as a limitation, potentially reducing the effectiveness of AI-driven consultations. However, others saw the use of AI as an advantage, as it might provide a neutral and judgment-free environment for discussing personal or sensitive health concerns. In this sense, rather than creating a sense of detachment, AI-driven apps could offer a new and alternative way of engaging with health-related issues.

*The real loss would be the human element—the fact that we would be handing over this work to something that isn’t human.*

[Male #5]

*If I imagine consulting a human—a doctor or a nutritionist—they can provide reassurance and emotional support. I’m not sure a chatbot could offer the same kind of relational interaction.*

[Male #11]

*There are now online platforms offering certified psychotherapy sessions. On one hand, these services involve professionals, but on the other, the screen can be both a barrier and a tool that helps people open up.*

[Female #2]

### Perceived Risks

#### Overview

The adoption of AI-driven mHealth apps could bring various uncertainties that would need to be addressed before these technologies can become widely implemented with minimal risks to users. Among the most prominent concerns raised during the focus group discussions were issues related to data privacy, the potential need for human oversight, and broader knowledge gaps regarding AI’s role in health care.

*The risk is that the app might promote behaviors that are not suitable for all individuals or body types and fail to acknowledge differences.*

[Male #12]

*The issue of privacy is significant. How much data do I have to provide to get a personalized AI-powered service? That’s something I worry about.*

[Female #2]

#### Subtheme 1: Privacy and Data Security

A significant portion of participants voiced concerns regarding the collection and management of sensitive personal data, particularly health-related information. They questioned the extent of the data being requested, the entities responsible for processing it, and the specific purposes for which it might be used. Some participants advocated for state or governmental oversight of data management, believing that public regulation would provide greater protection than private organizations. However, a shared expectation across the discussions was the need for transparency in data handling. Participants emphasized that sensitive health data should not be exploited for commercial purposes, such as market profiling.

*Speaking of privacy, I remember an incident—last year, I think—in the United States. In some states where abortion had been made illegal, women using period-tracking apps found out that their sensitive data had been sold. That’s definitely worrying.*

[Female #3]

*In my opinion, the data shouldn’t be, let’s say, managed by private companies, but by the national healthcare system. So, basically, the management should be as state-run as possible.*

[Female #2]

*Privacy is a big concern. You hear people talking about it—what happens to our data when we use these apps?.*

[Female #1]

#### Subtheme 2: Human Supervision and Control

Many participants agreed that AI-driven mHealth apps should be envisioned not as standalone tools but rather as supplementary aids under the guidance of health care professionals. These apps were viewed as potentially useful in assisting specialists but not as substitutes for expert judgment. The presence of a medical professional was regarded as crucial to ensure the accuracy of recommendations, assist users in interpreting AI-generated advice, and safeguard sensitive data.

*Losing the human element is dangerous, especially in healthcare. I would feel more comfortable talking to a doctor who can guide me through my health journey.*

[Male #5]

*As long as there is human oversight, AI in these apps is fine.*

[Female #3]

#### Subtheme 3: Uncertainty and Knowledge Gaps

The discussions also highlighted various uncertainties and knowledge gaps surrounding AI technology and its integration into health care applications. Participants expressed uncertainty about how well AI could handle the complexity of health-related issues, particularly when distinguishing between everyday concerns and more serious conditions. They also raised unexpected but resonant concerns about the long-term implications of widespread AI adoption, such as its environmental footprint.

*For very basic issues, AI might work. But where is the boundary between what a chatbot can handle and what requires a real doctor’s visit?*

[Female #1]

*I think the risk of errors decreases over time, but another concern that hasn’t been mentioned is the environmental impact. AI consumes a huge amount of energy, and if we start using it for everything, including these apps, we might face sustainability issues.*

[Male #7]

## Discussion

This section presents the principal findings regarding individuals’ attitudes toward AI-driven mHealth apps, followed by a discussion contextualizing these results within existing literature. Both consistencies with previous work and novel insights from the study are highlighted, followed by an examination of the limitations and suggestions for future research directions.

### Key Findings

This study explored how potential users perceive and anticipate AI-powered mHealth applications, identifying key factors that could influence usability, reliability, emotional interaction, and perceived risks. The findings suggest that participants associated personalization, accessibility, and data monitoring with significant advantages, whereas primary concerns centered around privacy, data security, and the reliability of AI-generated information. In addition, aspects such as communication style and the role of human interaction elicited mixed opinions, highlighting an ambivalent attitude toward adopting these technologies. These attitudes seemed closely linked to past experiences, with factors like monitoring and cost being evaluated through familiarity with traditional mHealth apps, while aspects like speed and automation were framed within broader AI-related expectations.

Participants appreciated the potential accuracy and adaptability of AI-driven mHealth apps, recognizing their ability to enhance user experience through tailored recommendations and real-time updates. However, preferences regarding AI’s communication style varied: while some users favored a clear and professional tone, others found a more friendly and engaging approach to be more effective. To accommodate this diversity, app developers could consider integrating customizable tone settings or adaptive algorithms that adjust communication style based on user preferences or behavior [30,31]. Furthermore, despite acknowledging the accessibility and cost-effectiveness of these technologies, some participants expressed concerns about the quality and depth of AI-provided responses compared to professional consultations. This highlights communication style as a key design consideration and suggests that tone flexibility may improve user comfort and engagement.

Reliability emerged as another critical issue. While some participants viewed technological advancements as a positive factor, many emphasized the need to verify AI-generated information before fully relying on it. This concern reflects broader uncertainties about AI’s role in health management, balancing its potential as a supportive tool with skepticism about its ability to replace human judgment, particularly in complex or sensitive situations.

Given the novelty of AI-driven mHealth apps and the limited direct experience with these tools, participants’ attitudes were largely shaped by past interactions with traditional mHealth apps for certain factors (eg, monitoring and cost) and by their broader knowledge or experiences with AI for others (eg, speed and automation). This suggests that their perceptions stem from an attempt to integrate these 2 reference points. Future research could further investigate how these general attitudes develop, whether certain factors weigh more heavily in shaping acceptance or rejection, and what individual differences contribute to the ambivalence observed in responses to these emerging technologies.

### Relation to Existing Literature

While very few studies have directly examined users’ perceptions of the risks and benefits of AI-driven mHealth apps [8,32], existing research has explored related aspects by focusing either on AI in health care more broadly or on the characteristics and user expectations of traditional mHealth apps. Some findings from these studies are consistent with those of this study, whereas others reveal key differences that emerge when AI is integrated into mHealth solutions.

Usability emerges as a key factor influencing attitudes toward AI-driven mHealth apps, with personalization playing a particularly strong role in fostering positive perceptions. Participants anticipated AI’s ability to offer tailored recommendations, real-time feedback, and enhanced accessibility—features that, when combined with the affordability of mHealth solutions (eg, diet planning or personalized fitness programs), were imagined to make AI integration particularly appealing. These findings align with existing literature on user engagement in mHealth, which emphasizes customization as a critical driver of adoption [9,27,28]. However, this study also reveals a degree of ambivalence: while many users envisioned AI as efficient and convenient, others expressed uncertainty about its communication style and interaction patterns. This variability in perception suggests that attitudes are shaped not by direct experience with these technologies but by an attempt to extrapolate from existing knowledge of AI and mHealth.

The theme of innovation and reliability elicited mixed reactions, with attitudes varying significantly based on users’ previous knowledge of AI and mHealth apps rather than the actual use. Some participants imagined AI as a natural progression in digital health care, viewing it as a promising tool for health management—an attitude that may reflect a form of pro-innovation bias [33], where technological advancements are perceived as inherently beneficial. Conversely, others remain skeptical, particularly regarding the accuracy and reliability of AI-generated recommendations. This skepticism appeared more connected to general concerns about AI technology rather than direct experience with AI-driven mHealth apps [12,21], suggesting that participants’ attitudes reflected pre-existing beliefs about AI’s trustworthiness rather than firsthand interactions [10,34,35].

Reliability concerns also intersected with how users perceive mHealth apps as a monitoring tool. While some individuals expected AI-driven mHealth apps to enhance traditional tracking features through advanced data analysis and personalization, others remained cautious about their role in delivering medical advice. The qualitative data suggests that trust in AI-driven recommendations depended more on participants’ general exposure to AI-based tools rather than direct usage of AI-driven mHealth apps. Participants familiar with AI-powered technologies appeared more open to their guidance, whereas those with limited knowledge expressed greater hesitation—a dynamic consistent with patterns observed in AI adoption literature [19,21,36]. These findings reinforce the notion that trust in technology is not only shaped by technical performance but also by users’ broader conceptualizations, expectations, and personal experiences of AI in different contexts.

The psychological and emotional impact of AI-driven mHealth apps presents a particularly nuanced dynamic, as the same features were imagined to elicit contrasting reactions among different users. One central factor in this divergence was human-AI interaction, particularly in hypothetical scenarios where AI replaced direct human engagement. Some users expected AI-based coaching to reduce social pressure, finding it less intimidating than interactions with human professionals. For them, AI was envisioned as a judgment-free space for self-improvement, enhancing feelings of control and autonomy—a benefit highlighted in studies examining patient-clinician dynamics [37]. However, others express discomfort with the absence of human empathy, speculating that AI may fail to provide the emotional support and nuanced understanding required in sensitive health-related contexts, as noted in research on AI’s limitations in therapeutic settings [38].

This tension is closely connected to the broader discussion on human supervision and control. Participants who are skeptical of AI’s role in health care emphasize the need for human oversight, particularly in situations where AI-generated advice could have serious implications. This perspective aligns with frameworks proposing hybrid human-AI systems [39], and is supported by findings showing concerns about fully automated health solutions in the absence of clear human intervention mechanisms [40]. These observations collectively reinforce the perspective that AI-driven medical solutions were imagined as more readily accepted as assistive tools rather than autonomous decision-makers [6,12].

Privacy concerns and data governance emerged as dominant themes, revealing participants’ surface-level awareness of legal risks surrounding sensitive health data management. While users expressed concrete anxieties about data access controls [18], storage security protocols, and risk-benefit tradeoffs of AI convenience versus potential breaches, their understanding of specific regulatory frameworks remained limited. This highlights the need for clearer communication around data governance. Developers could help bridge these knowledge gaps through evidence-based user education strategies, such as (1) interactive onboarding tutorials with just-in-time explanations, (2) “privacy nutrition labels” for transparent policy summaries, and (3) participatory consent processes that visualize data flows—all proven to enhance comprehension and trust in health AI contexts [41,42]. Critically, these approaches move beyond static disclosures to dynamic, user-centered designs that align with real-world decision-making needs [42]. These findings mirror broader literature identifying privacy paradoxes in digital health adoption [14], where expressed concerns often outweigh practical protective behaviors. Notably, these apprehensions coexisted with broader skepticism about AI’s reliability in health care contexts, leading some users to stress the perceived necessity of human supervision, framing it as both a quality control mechanism and an ethical safeguard. This concern permeated multiple discussion topics in the findings, suggesting it represents a core barrier to trust in AI-driven health care tools.

This landscape of uncertainty spanned multiple dimensions—both technical and conceptual. Participants questioned not only how AI-driven systems function algorithmically but also how they comply with existing regulations, what monitoring systems are in place, and who holds accountability for decision-making. Notably, these knowledge gaps extended beyond core functionality to include unexpected concerns, such as the potential environmental impact of AI infrastructure. Although mentioned only once in focus groups, this issue quickly resonated with other participants, suggesting that ecological considerations (eg, energy consumption of AI models [43]) may subconsciously influence perceptions of mHealth solutions, even among users without direct technical knowledge. Such broad uncertainty, encompassing both technical and nontechnical factors, frequently characterizes public reception of emerging health technologies [18], particularly when transparency about decision-making processes appears insufficient. These concerns frequently translated into demands for clearer accountability frameworks [44] and verifiable regulatory compliance [45], as fundamental requirements for trust-building.

The analysis reveals that attitudes toward AI-driven mHealth apps stem from multiple interconnected factors. These include: (1) theoretical expectations about technical app characteristics (eg, usability and personalization features), (2) individual tendencies to trust or distrust technology, and (3) broader ethical-legal concerns. Crucially, these factors do not operate in isolation but interact dynamically, creating unique perceptual configurations for different users. This complex interplay ultimately determined the spectrum of observed responses—from enthusiastic anticipation to cautious skepticism or outright rejection.

### Limitations

While this study provides valuable exploratory insights, several limitations should be acknowledged. First, the gender imbalance within the sample (12 men vs 3 women) may have influenced the findings. Although existing evidence refers separately to mHealth apps and to AI apps in health care, studies suggest that gender can affect attitudes toward these technologies, and similar dynamics may also apply to AI-driven mHealth apps. For example, women tend to report higher health literacy and greater concerns about privacy, while men may place more emphasis on response efficacy and perceive these technologies as easier to use [46,47]. This imbalance may have shaped the salience of certain themes—such as emotional interaction, communication style, and privacy concerns—highlighting the importance of considering gendered perspectives when interpreting qualitative findings [48]. Future research should therefore aim to achieve more balanced gender representation and could more rigorously explore gendered dimensions by incorporating stratified sampling strategies, gender-sensitive interview protocols, or comparative analysis frameworks designed to detect gender-specific themes [49].

Regarding the age distribution, the study focused on young adults (mean age: 27 years; range: 25‐34 years), a demographic that represents a particularly relevant target group for AI-mHealth given their high engagement with digital health tools and greater trust in AI technologies [22-24]. However, this choice necessarily limits the generalizability of findings to other age groups, whose needs, digital literacy, and expectations may differ [50,51]. Future research could explore these age-related differences through targeted sampling and quantitative approaches. Finally, as an exploratory qualitative study, the methodological approach is inherently limited by potential group dynamics, such as dominant voices or social desirability [52]. Future research could benefit from robust mixed methods designs to address some of the limitations mentioned earlier.

### Conclusions and Future Directions

This study offers valuable insights into users’ attitudes toward the potential integration of AI in mHealth apps, focusing on key advantages such as personalization, accessibility, cost-effectiveness, and their potential as monitoring tools that leverage AI for tailored recommendations. The immediacy and affordability of such apps were seen as notable benefits, suggesting that they could make health management more accessible compared with traditional professional consultations. However, concerns about data management, transparency, and users’ understanding of these technologies emerged as significant barriers. Given the novelty of AI-driven mHealth apps, many participants formed opinions based on general perceptions of AI or traditional mHealth apps rather than direct experience, highlighting a broader uncertainty about their functionality and reliability.

Ambivalent attitudes were particularly evident regarding the perceived reliability of AI-generated recommendations, the need for human supervision, and the psychological impact of AI interactions. These factors elicited mixed reactions, with responses largely influenced by previous knowledge, pre-existing opinions about AI, and attitudes toward digital health tools. In addition, certain themes, although not central to the main discussion, warrant further exploration. For instance, a pro-innovation bias was observed in some users, indicating a tendency to view technological advancements as inherently beneficial. Furthermore, concerns about the environmental impact of AI systems—though mentioned only briefly—resonated with a broader segment of participants, highlighting a potential influence on consumer attitudes.

Developers could focus on highlighting the positive aspects that emerged in this study, such as the increased personalization options offered by AI-driven mHealth apps, their cost-effectiveness, and their strong monitoring capabilities for health data. At the same time, they should address potential risks or concerns, such as enhancing the clarity and transparency of data management practices, particularly around privacy, and ensuring systems of periodic human supervision. Furthermore, developers could provide greater customization for those ambivalent factors by allowing users to select the type of interaction with the AI, adapting the intensity of interactions to suit personal preferences—for example, adjusting the tone to be more formal or informal, scientific or motivational, and so on.

Future research should address the limitations of this study and explore these underlying factors in greater depth. Using quantitative or mixed-methods approaches could provide deeper insight into the psychological and behavioral drivers that influence attitudes toward the integration of AI in mHealth apps. This would ultimately guide the development of more user-centered and ethically responsible strategies for these technologies, ensuring a vision that not only protects and supports end users in the most comprehensive way possible, but also addresses the delicate nature of health-related matters. Furthermore, as AI-driven mHealth apps become more widely available, longitudinal studies tracking user perceptions before and after direct experience with such technologies could offer valuable insights into the evolution of attitudes toward these technologies over time [27,28]. Given the novelty of these technologies, such contributions can make a real difference, both in the academic landscape and in practical terms, by helping design safer, more effective, and widely accepted AI-driven mHealth apps that truly meet the needs of end users.

## Abbreviations

AI: artificial intelligence
mHealth: mobile health
